# Pinch-off mechanism in double-lateral-gate junctionless transistors fabricated by scanning probe microscope based lithography

**DOI:** 10.3762/bjnano.3.91

**Published:** 2012-12-03

**Authors:** Farhad Larki, Arash Dehzangi, Alam Abedini, Ahmad Makarimi Abdullah, Elias Saion, Sabar D Hutagalung, Mohd N Hamidon, Jumiah Hassan

**Affiliations:** 1 Department of Physics, Universiti Putra Malaysia, 43400 Serdang, Selangor, Malaysia; 2 Institute of Microengineering and Nanoelectronics (IMEN), Universiti Kebangsaan Malaysia, 43600 Bangi, Selangor, Malaysia; 3 School of Materials and Mineral Resources Engineering, Universiti Sains Malaysia, 14300 Nibong Tebal, Penang, Malaysia,; 4 Functional Devices Laboratory, Institute of Advanced Technology, Universiti Putra Malaysia, 43400 Serdang, Selangor, Malaysia

**Keywords:** AFM nanolithography, junctionless transistors, pinch-off, scanning probe microscope, simulation

## Abstract

A double-lateral-gate p-type junctionless transistor is fabricated on a low-doped (10^15^) silicon-on-insulator wafer by a lithography technique based on scanning probe microscopy and two steps of wet chemical etching. The experimental transfer characteristics are obtained and compared with the numerical characteristics of the device. The simulation results are used to investigate the pinch-off mechanism, from the flat band to the *off* state. The study is based on the variation of the carrier density and the electric-field components. The device is a pinch-off transistor, which is normally in the *on* state and is driven into the *off* state by the application of a positive gate voltage. We demonstrate that the depletion starts from the bottom corner of the channel facing the gates and expands toward the center and top of the channel. Redistribution of the carriers due to the electric field emanating from the gates creates an electric field perpendicular to the current, toward the bottom of the channel, which provides the electrostatic squeezing of the current.

## Introduction

The fabrication of transistors without junctions and a doping concentration gradient has been introduced recently as a potential way to overcome the major obstacles in ultrascaled transistors [1–2]. Accordingly, based on simulation studies, performance estimates of junctionless transistors (JLTs), quantum ballistic transport, and novel structures such as bulk planar junctionless transistors (BPJLTs) have also been investigated [3–5]. The idea behind the JLTs, or pinch-off transistors [6], is to simplify the source/drain engineering by removing the conventional junctions, and at the same time, facilitating the scaling of the transistors. The structures of proposed JLTs utilize a thin channel with homogeneous doping polarity and high doping concentration across the source/drain and the channel. High doping concentration can provide a higher value of current for such a thin channel, but at the same time causes an unavoidable scattering effect and subthreshold swing (SS) fluctuation. The latter case can justify new experiments with low doping concentration for JLTs.

The fabrication of low-doped single-lateral-gate (SG) and double-lateral-gate junctionless transistors (DGJLT) by scanning probe microscope based lithography (SPL) via local anodic oxidation (LAO) was reported previously [7–9]. The experimental characteristics were also investigated and single-gate and double-gate structures were compared [10]. The principle of SPL on silicon-on-insulator (SOI) was described for the first time by Snow et al. [11]. Subsequent results by this technique are presented in the references [12–13]. In fact, fabrication of nanostructures by SPL and particularly by using atomic force microscope (AFM) nanolithography has been developed with prominent results, and similar structures have been fabricated and experimentally characterized [14–15]; however, the lack of sufficient explanation for the behavior of these structures is still an interesting issue. Moreover, similar structures have never been investigated numerically, or ever used as pinch-off devices.

In this work, we investigate some of the influential factors on SPL by AFM nanolithography, to obtain the optimized parameters for fabrication of the DGJLT. We also used 3-D TCAD simulations to investigate the principles of the DGJLT in the *off* state. We investigate the electron/hole density distribution and electric-field components along the channel and the source/drain extension in order to obtain a better understanding of the device performance through the pinch-off mechanism.

## Methodology

**Figure 1:**
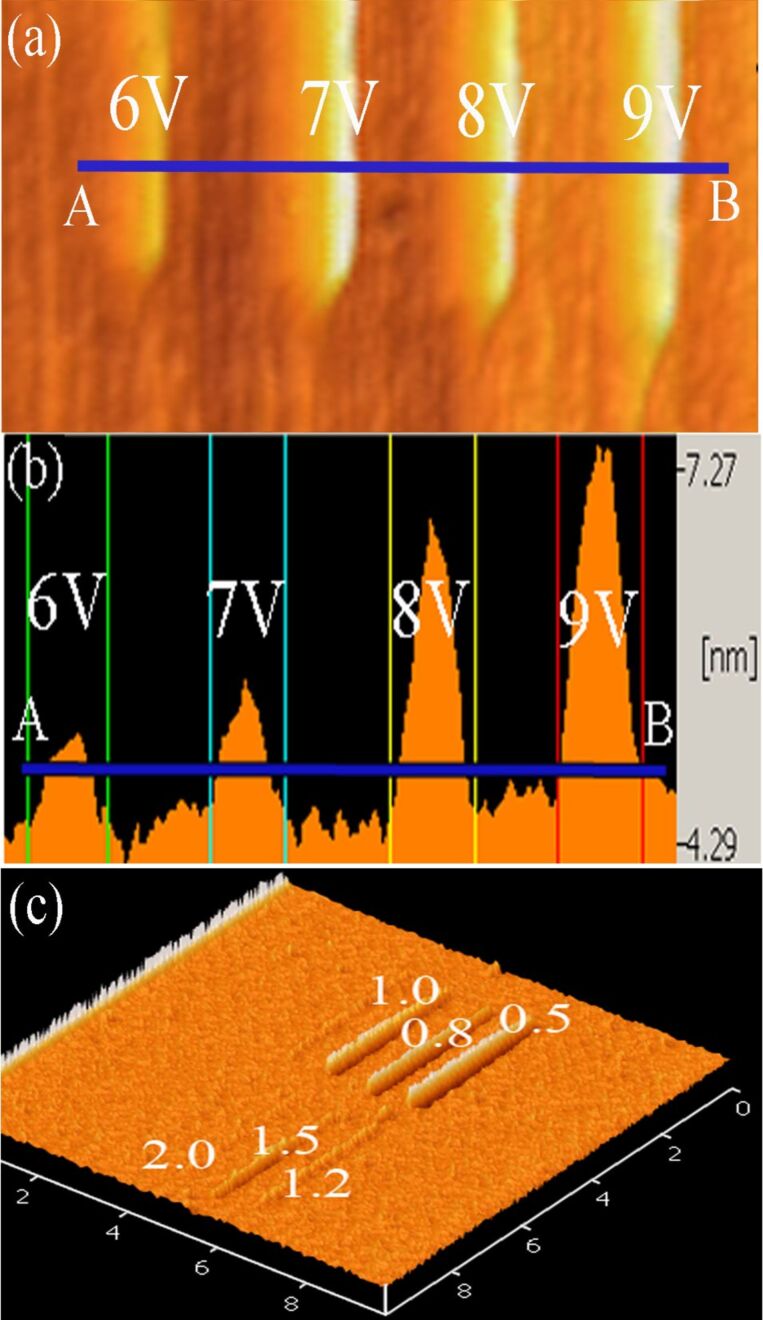
(a) AFM topographic images depicting a series of oxide protrusions produced by applying various voltages in the range 6–9 V. (b) Cross-section profiles along AB lines indicated in (a). (c) Effect of writing speed at a constant voltage of 9 V.

The DGJLT structure was physically fabricated by using the local anodic oxidation (LAO) process and two wet-chemical-etching processes. Pre-oxidation sample preparation steps, fabrication method, and parameters were elaborately mentioned in our previous works [8,10]. LAO by AFM nanolithography was carried out on a lightly doped (10^15^ cm^−3^) p-type (100) SOI wafer with top silicon thickness of 100 nm and a 145 nm buried oxide (BOX) thickness with a resistivity of 13.5–22.5 Ω cm [16], by using scanning probe microscope (SPM) (SPI3800N/4000). The buried oxide layer in the SOI wafer was used as an insulator between the device and the handle silicon wafer, and also as the etch-stop in the wet-etching process. All predesigned oxide masks were fabricated in contact mode employing a Cr/Pt coated tip with a force constant of 0.2 N m^−1^ and a resonance frequency of 13 kHz. The room humidity (RH) was controllable from 50% to 80% with an accuracy of 1%. At a constant RH and contact force, the AFM tip voltage and writing speed (exposing time) are important factors to determine the size of the oxide patterns. Figure 1a and Figure 1b show that oxide protrusions were produced on the hydrogen passivated silicon surface with various voltages from 6 to 9 V at a constant writing speed of 1 µm/s, at 65% RH, and in contact mode. The effect of different writing speeds at constant voltage of 9 V is shown in Figure 1c. The optimized oxidation parameters correspond to a voltage of 9 V on the tip, with a speed of 1 µm/s, with the RH in the range of 65–67%.

**Figure 2:**
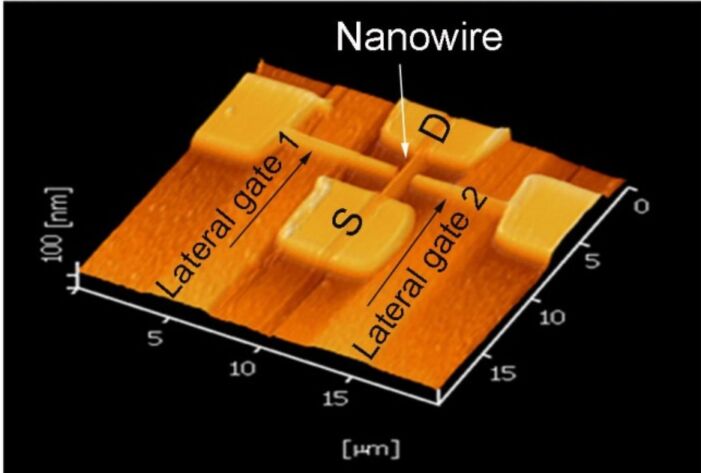
AFM topography image of the final structure.

After patterning of the oxide mask, the first step of wet chemical etching was carried out with a solution of 30 wt % potassium hydroxide (KOH) saturated with 10 vol % isopropyl alcohol (IPA) at 63 °C for 20 s, in order to remove the unmasked Si layer. IPA was used as an initiator to improve the cleaning process: it reduces the etch rate, improves the surface roughness and makes the etching process more controllable [17]. This step of device fabrication is very significant and high accuracy and precision is required in order to obtain a smooth and uniform surface. The final step of the device fabrication is the etching of the silicon oxide, which allows the removal of the oxide mask. The silicon oxide etch was performed with diluted hydrofluoric acid (H_2_O/HF 100:1). The immersion time of the sample in the HF solution was about 16 to 18 s. Figure 2 shows the AFM topography image of the DGJLT after two steps of successive etching.

Final parameters of the fabricated structure, with the best gate symmetry and reproducibility of dimensions that we achieved, are given in Table 1. According to the fabrication method, the whole structure has the same thickness (100 nm) and doping concentration of 10^15^ cm^−3^ throughout the channel, source/drain, and lateral gates. The lateral gates are located 100 nm away from the channel region.

**Table 1:** Fabricated/simulated device parameters.

	width	thickness	length

nanowire	100 nm	100 nm	4.2 µm
S/D/Gates pad	5 µm	100 nm	5 µm
lateral gates	200 nm	100 nm	4.5 µm

**Figure 3:**
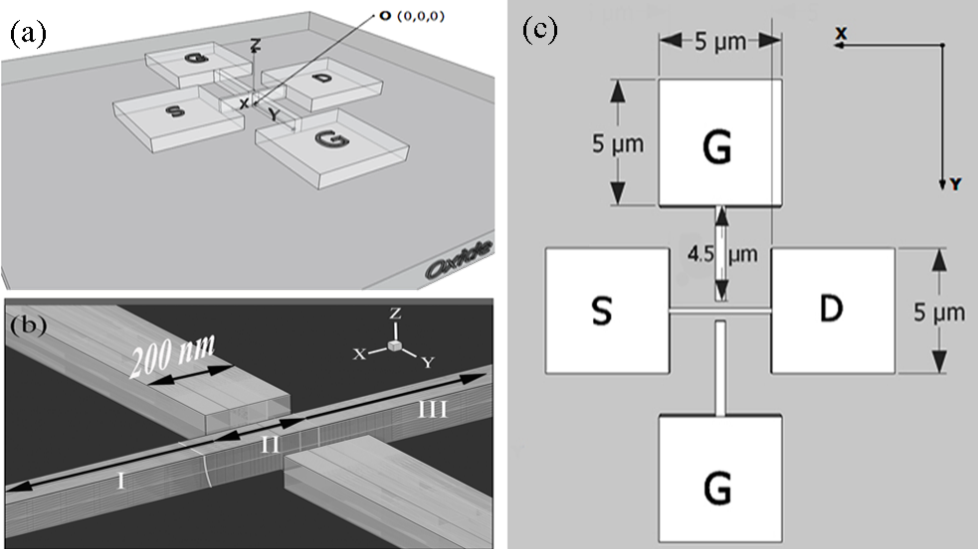
(a) Isometric view of the simulated device. (b) Blow up view of the gated area and different nanowire zones. (c) Top view of the simulated device.

In this paper, 3-D simulations of the DGJLT were carried out by the Sentaurus 3-D device simulator [18]. All parameters in the simulation process are analogous to the fabricated device as mentioned in Table 1. The isometric view of the simulated device structure is schematically presented in Figure 3a. In accordance with the fabricated structure, the simulated structure also consists of a nanowire, two wires as lateral gates, and four square pads as source, drain, and gate contacts. The complete structure sits on a 145 nm ideal oxide. The origin in all simulation results is considered to be in the center at the bottom of the channel with the BOX interface. According to the material of the tip used in the experimental measurements (Tungsten), the work function of the contacts is taken to be 5.12 eV in all simulation steps. The simulations were carried out by using the hydrodynamic model. The doping-dependent Masetti mobility model, which incorporates the high-field-saturation Canali model, is used in order to examine the high-electric-field effect. Doping-dependent Shockley–Read–Hall recombination-generation [19] was applied in order to consider the leakage current and recombination through deep defect levels in the gap. As is shown in Figure 3b, for ease of explanation in future references, the nanowire between the source and the drain contacts is divided into three different zones, labeled as X_I_, X_II_, and X_III_, with lengths of 2 µm, 200 nm, and 2 µm, respectively. In Figure 3c, the top view of the simulated structure is shown schematically. Here, the dimensions of the source, drain, and gate pads, as well as lateral gate length are demonstrated.

## Results and Discussion

**Figure 4:**
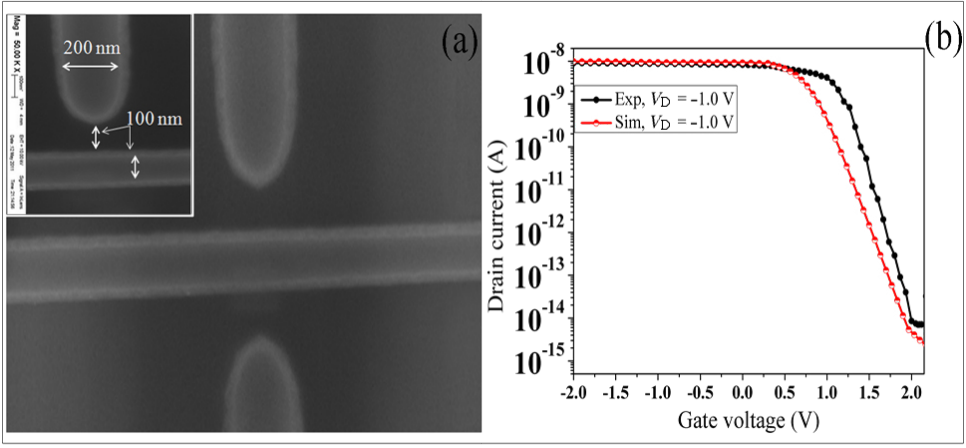
(a) SEM image of the gated area. (b) Experimental measurement and simulated transfer characteristics of DGJLT at *V*_DS_ = −1.0 V.

A scanning electron microscope (SEM) image of the gated area of the device with the best gate symmetry is shown in Figure 4a. Transfer characteristics (*I*_D_–*V*_G_) from experimental measurements and 3-D TCAD simulation results for DGJLT are depicted in Figure 4b. The electrical characteristics of the device were measured by the HP4156C semiconductor parameter analyzer (SPA, Agilent) at room temperature. It should be noted that the measurement setup has four highly accurate source/monitor units (SMUs) and is designed for Kelvin connections. Four lakeshore Tungsten tips with 3 µm radius were used with the SMUs to measure the electrical characteristics of the device. An overall good agreement is found between measurement and simulation results. The on-currents of simulated results and experimental measurement are of the same order of magnitude. However, the experimental *off* state current agreement cannot be determined, due to the limitations of our measurement instrument. A small variation in threshold voltages (*V*_th_) between the simulation and experimental curves can be illustrated by the presence of fixed interface charge, work function differences, or both [20].

It should be mentioned that, during the KOH anisotropic etching process, very complicated three-dimensional structures based on the etchant concentration, temperature, and angle between the silicon surface and the mask [21] are likely to be created. However, in all simulation steps the ideal initial cubic shape is considered, for simplicity. We believe that, according to the device dimensions in the simulation study, considering the exact shape of the device edges can only slightly affect the critical characteristics of the devices.

Figure 4b indicates that, at zero gate voltage, the device is in its *on* state when it is biased by a nonzero drain–source voltage. A sufficient positive bias applied to the lateral gates depletes the region under the gates and cause an *off* state. The transfer characteristic curves also show that the device has on–off ratio of 10^6^ and 10^7^ for the fabricated and simulated device between *V*_G_ = 0 V and *V*_G_ = 2 V, respectively. In the case of accumulation MOSFETs (AMOSFETs) and JLTs (gated resistors), the depletion of the channel region is in the *off* state at *V*_G_ = 0 V, caused by the work-function difference between the gate material and the highly doped channel [2,20]. As a result, in all fabricated devices a gate bias voltage equal to the work-function difference between channel and the gate is required to achieve a flat-band condition. It is worth noting that the JLT is principally a gated resistor that is normally an *on* device at *V*_G_ = 0 V [22]. When zero gate bias is applied to DGJLT, the entire channel region is neutral (i.e., not depleted), and the device is in a flat-band condition. The similar and low doping concentration of the channel and the gates eliminates the effect of a work-function difference between the channel and the gates, which provides the flat-band condition at zero gate voltage.

According to the equation proposed in [10] the saturation drain current of DGJLTs is given by,

[1]
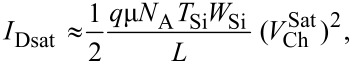


where *W*_Si_ is the width of the silicon, *T*_Si_ is the thickness of the silicon, *N*_A_ is the doping concentration, *L* is of the order of *L*_G_, and 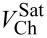 is the effective channel voltage, which obeys


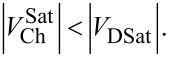


In JLTs, the operation is designed to start from the *off* state at zero gate voltage, and be driven into the *on* state by means of a proper gate voltage [5,23], but in a pinch-off device such as a DGJLT, the operation is implemented in the reverse direction, in order to force the device into the *off* state from the *on* state.

**Figure 5:**
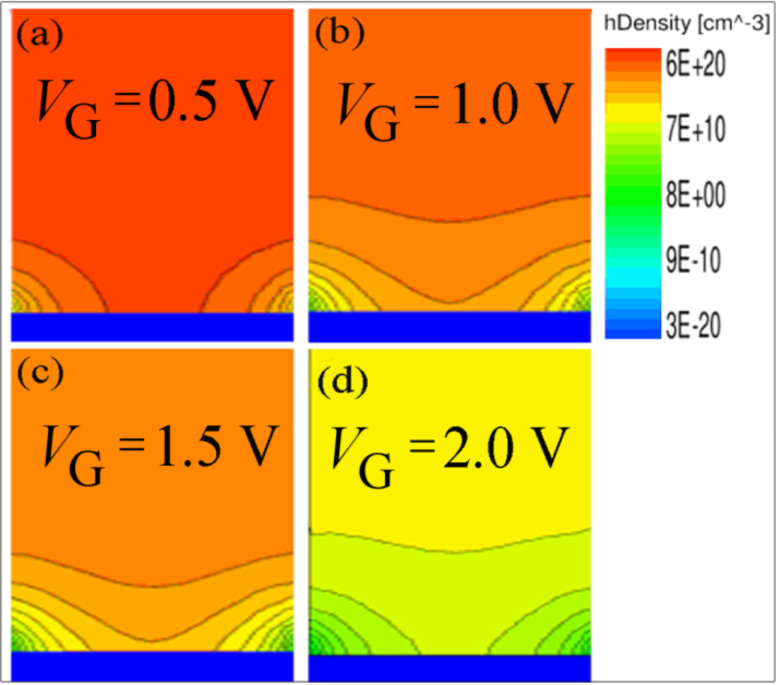
Hole concentration for a vertical cut of DGJLT at (*X* = −100 nm) for four different gate voltages (a) *V*_G_ = +0.5, (b) *V*_G_ = +1.0., (c) *V*_G_ = +1.5, and (d) *V*_G_ = +2.0 V. The contours present the depletion of carriers due to the gate effect.

The mechanism of depletion due to the lateral gate voltage can be demonstrated by the hole distribution in the channel. Figures 5a–d show the simulated results for the hole density distribution in the channel profile (*X*-cut) for different gate voltages from *V*_G_ = +0.5 to +2.0 V.

When the device is in the *on* state and a positive gate voltage is applying to the lateral gates, the channel starts to deplete, and at a sufficient positive gate voltage the channel is fully depleted and the device is in the pinch-off condition. For the DGJLT, due to the specific shape of the device and having only one interface with the BOX at the bottom, the undepleted (neutral) area is located in the center of the channel [10]. As is shown in Figure 5, the depletion starts at the bottom corner of the channel, facing the gates, and expands toward the center and the top of the channel.

**Figure 6:**
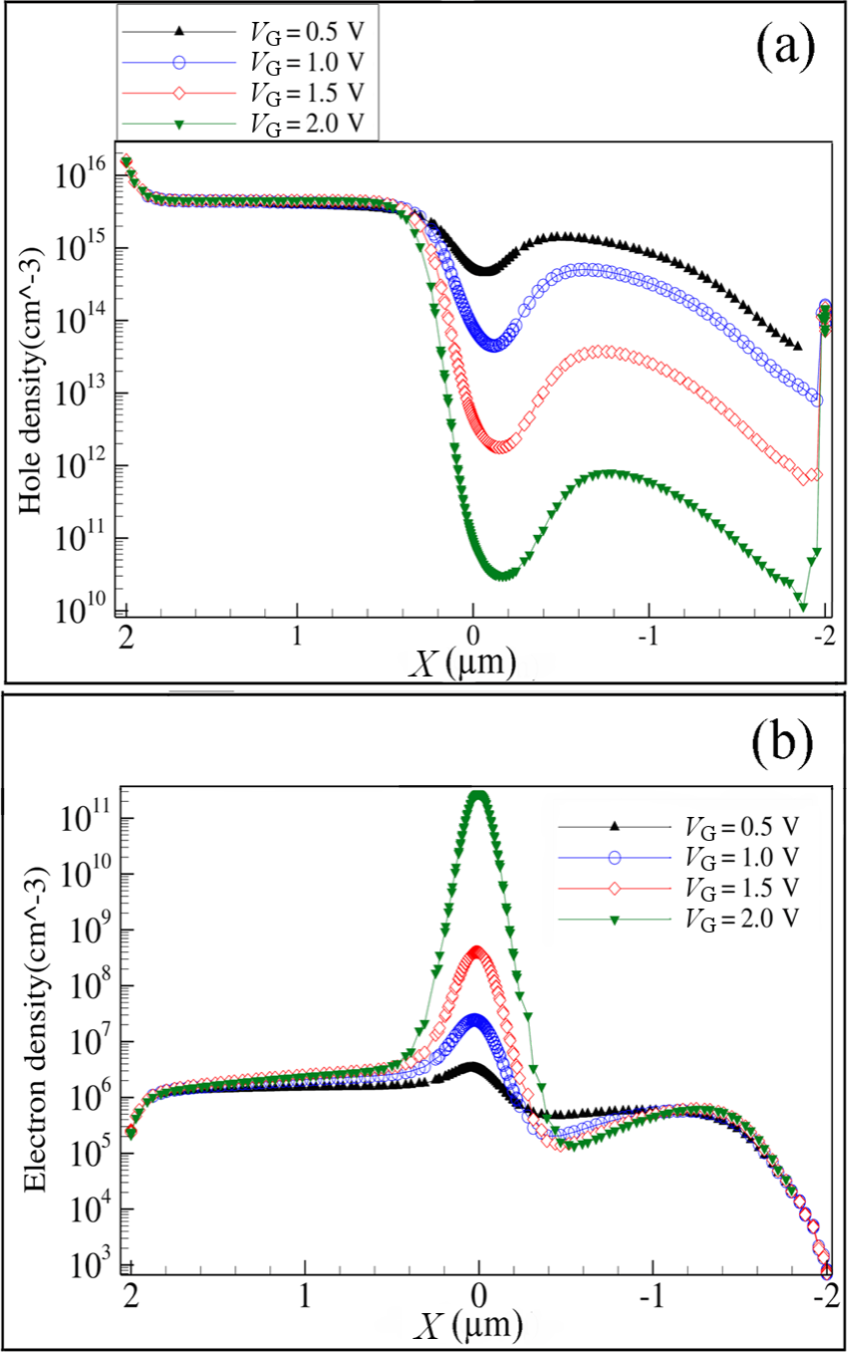
(a) Hole density and (b) electron density distribution as a function of position along a horizontal cross section, at four different gate voltages (0.5, 1.0, 1.5, 2.0 V), *V*_DS_ = −1.0 V.

Figure 6 shows the hole and the electron density distribution along the channel axis at four different positive gate voltages, from near flat-band to pinch-off state. As the graphs of the hole density distribution show, the main depletion starts from the drain side of the gate (X_II/III_ interface).

The holes in the channel are repelled by the gate voltages and swept up by the drain contact; therefore, the higher depletion rate occurs at the higher positive gate voltage (Figure 6a).

When the positive gate voltage increases, the electrons start to accumulate under the gated area, and the higher voltage provides more accumulated electrons in the channel. Since the electrons in the gated region are mostly absorbed by the positive gate voltage and simultaneously repelled toward the channel by the negative drain voltage, lower electron density occurs in the drain extension (Figure 6b).

**Figure 7:**
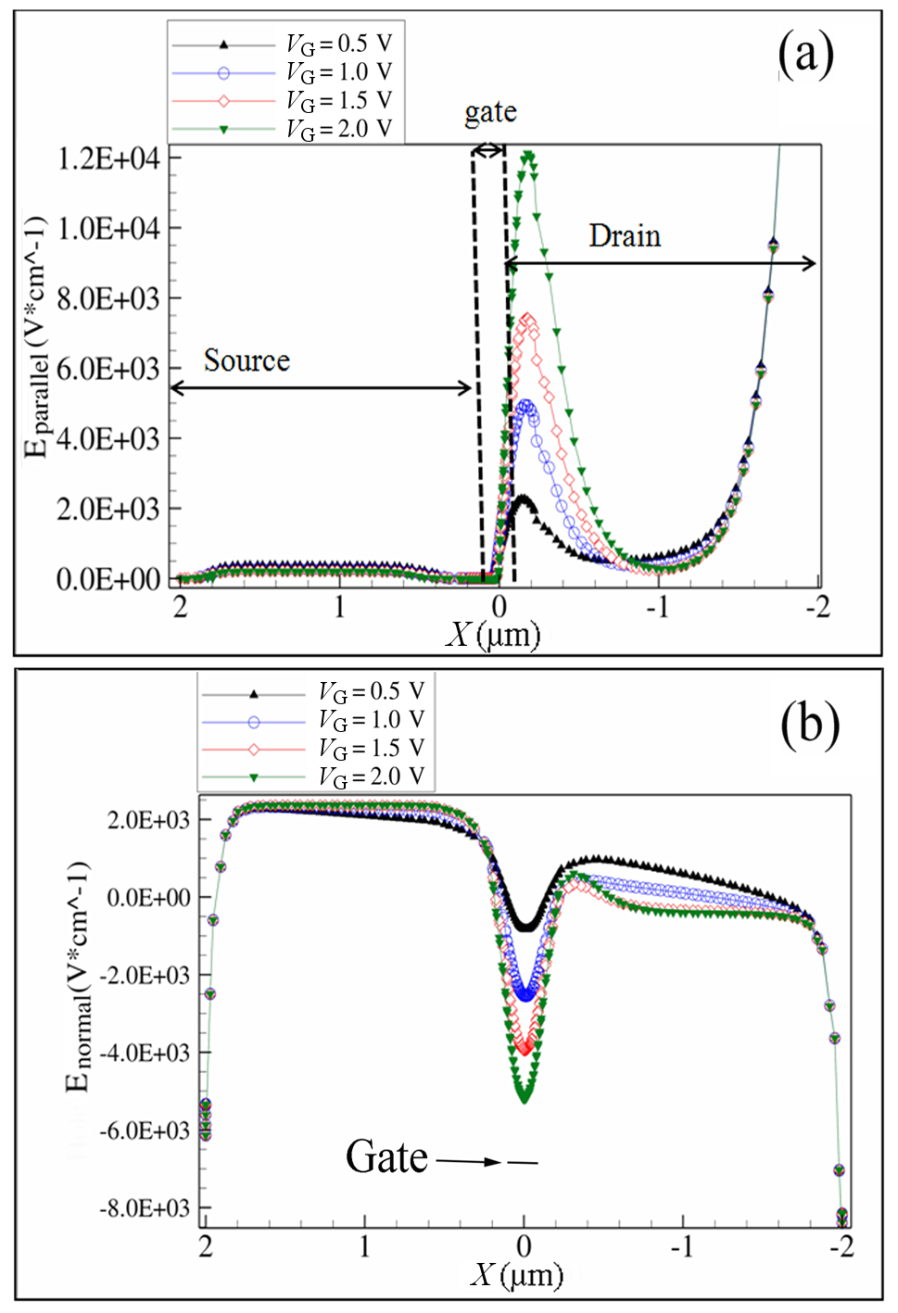
Simulated (a) parallel and (b) normal electric field along a horizontal cut line at the center of the channel (source side to drain side), *V*_G_ = 0.5, 1, 1.5, and 2.0 V, *V*_ds_ = −1.0 V.

The complete depletion mechanism of the device relies on the ability of the electric field of the lateral gates to deplete the channel of holes, and the incapability of the drain to supply an influential electron population into this area. In fact, the introduced electrons from the drain contact are really negligible compare to the hole density (Figure 6b). The accumulated electrons in the channel create an area of higher electron potential energy. This potential difference establishes an electric field toward the zone X_I_ and provides a barrier against the holes passing through the channel from the source to the drain, which facilitates the pinch-off effect in the channel. Figure 7a and Figure 7b show the electric field parallel and perpendicular to the current flow at four different gate voltages from the flat-band to the pinch-off state. Wherever the accumulated electrons are higher (Figure 6b), due to the increased positive gate voltage, the components of the electric field are stronger. As the normal component of electric field indicates, and according to the mechanism of depletion (from the bottom to the center and top of the channel), a redistribution of the accumulated electrons and depleted holes provokes an electric field toward the bottom of the channel (Si/BOX).

Figure 7 indicates that the peak of the electric field is located at the area of lowest hole concentration, which confirms the normal behavior of JLTs [24]. It is worth noting that the region of high electric field is located in the drain extension of the DGJLT outside of the gated region, since current blocking is caused by pure electrostatic pinch-off effect over the channel. This is in contrast to conventional junction transistors in which the peak of the electric field is located in the channel region, right next to the metallurgical junction [23]. As a result, the influence of the drain electric field on the channel region of DGJLT is much smaller than in conventional transistors (inversion mode). The configuration of the electric field, in a direction perpendicular to the current, indicates that the minimum value of the normal electric field is below the threshold voltage, due to the formation of the electrically neutral conducting wire below the threshold voltage between the source and the drain. By increasing the gate voltage above the threshold voltage, the carriers are affected by a large electric field normal to the current flow. This is completely opposite to the case of accumulation-mode (AM) devices in which the highest electric field appears in the channel when the device is turned on [20]. By increasing the drain voltage, a high electric field in the drain extension creates a depletion area in zone X_III_. At sufficiently high electric field, full depletion of the nanowire in the drain extension acts as a buffer and prevents the drain electric field from propagating into the channel. This mechanism reduces the channel modulation effects and at high drain voltage, this barrier causes the saturation of current.

## Conclusion

We have presented fabrication of p-type DGJLTs using an unconventional method of scanning probe lithography and a numerical study of the same structures using 3-D TCAD simulation results. The analysis presented shows that the device can be considered as a simple FET device that includes no doping concentration gradient and no junction. Unlike the accumulation metal–oxide–semiconductor field-effect transistors (AMOSFETs) and junctionless nanowire transistors (JLNWT), in which the channel is depleted when zero gate voltage is applied to the device, the channel of the DGJLT is fully *on* at this gate voltage and the entire channel region is neutral. These simple devices are normally *on* transistors and the device can be forced to the pinch-off state as a result of full depletion of channel by the electric field created by the lateral gates.
